# *N*-(2,3-Dimethyl­phen­yl)-4-methyl-*N*-(4-methyl­phenyl­sulfon­yl)benzene­sulfonamide

**DOI:** 10.1107/S1600536812039773

**Published:** 2012-09-26

**Authors:** Shumaila Younas Mughal, Islam Ullah Khan, William T. A. Harrison, Muneeb Hayat Khan, Muhammad Nawaz Tahir

**Affiliations:** a Materials Chemistry Laboratory, Department of Chemistry, GC University, Lahore 54000, Pakistan; b Department of Chemistry, University of Aberdeen, Meston Walk, Aberdeen AB24 3UE, Scotland; c Questioned Documents Unit, Punjab Forensic Science Agency, Home Department, Lahore, Pakistan; d Department of Physics, University of Sargodha, Punjab, Pakistan

## Abstract

In the title compound, C_22_H_23_NO_4_S_2_, the dihedral angles between the dimethyl­phenyl ring and the two methyl­phenyl rings are 41.19 (15) and 20.50 (17)°; the dihedral angle between the methyl­phenyl rings is 48.11 (14)°. The C—N—S—C torsion angles are −87.6 (2) and 77.43 (18)°. The only possible directional inter­actions in the crystal are very weak C—H⋯π inter­actions and very weak π–π stacking between parallel methyl­phenyl rings [centroid-to-centroid separation = 4.010 (2) Å and slippage = 1.987 Å].

## Related literature

For a related structure, see: Mughal *et al.* (2012 ▶).
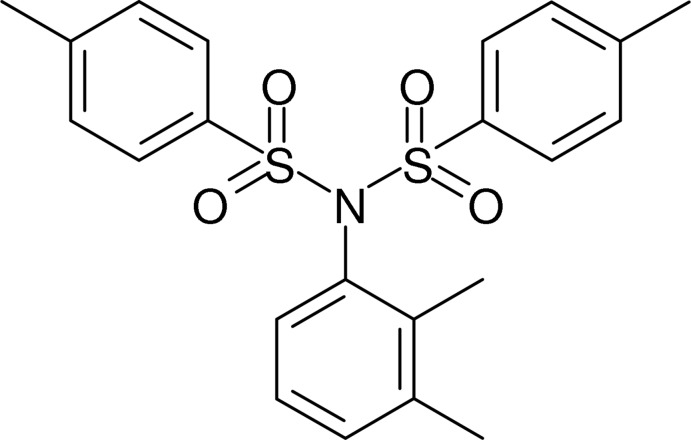


## Experimental

### 

#### Crystal data

C_22_H_23_NO_4_S_2_
*M* *_r_* = 429.53
Triclinic, 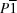

*a* = 7.5523 (8) Å
*b* = 8.4635 (9) Å
*c* = 17.5004 (17) Å
α = 103.282 (7)°
β = 91.659 (7)°
γ = 108.382 (7)°
*V* = 1026.89 (18) Å^3^
*Z* = 2
Mo *K*α radiation
μ = 0.29 mm^−1^
*T* = 296 K
0.32 × 0.23 × 0.12 mm

#### Data collection

Bruker APEXII CCD diffractometer
Absorption correction: multi-scan (*SADABS*; Bruker, 2007 ▶) *T*_min_ = 0.913, *T*_max_ = 0.966
16232 measured reflections
4569 independent reflections
2359 reflections with *I* > 2σ(*I*)
*R*_int_ = 0.068

#### Refinement

*R*[*F* ^2^ > 2σ(*F* ^2^)] = 0.055
*wR*(*F* ^2^) = 0.129
*S* = 0.96
4569 reflections
266 parameters
H-atom parameters constrained
Δρ_max_ = 0.24 e Å^−3^
Δρ_min_ = −0.30 e Å^−3^

### 

Data collection: *APEX2* (Bruker, 2007 ▶); cell refinement: *SAINT* (Bruker, 2007 ▶); data reduction: *SAINT*; program(s) used to solve structure: *SHELXS97* (Sheldrick, 2008 ▶); program(s) used to refine structure: *SHELXL97* (Sheldrick, 2008 ▶); molecular graphics: *ORTEP-3* (Farrugia, 1997 ▶); software used to prepare material for publication: *SHELXL97*.

## Supplementary Material

Crystal structure: contains datablock(s) global, I. DOI: 10.1107/S1600536812039773/xu5622sup1.cif

Structure factors: contains datablock(s) I. DOI: 10.1107/S1600536812039773/xu5622Isup2.hkl

Supplementary material file. DOI: 10.1107/S1600536812039773/xu5622Isup3.cml

Additional supplementary materials:  crystallographic information; 3D view; checkCIF report

## Figures and Tables

**Table 1:** Hydrogen-bond geometry (Å, °) *Cg* is the centroid of the C16–C21 ring.

*D*—H⋯*A*	*D*—H	H⋯*A*	*D*⋯*A*	*D*—H⋯*A*
C8—H8*B*⋯*Cg* ^i^	0.96	2.96	3.912 (4)	169

Symmetry code: (i) 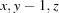.

**Fig. 1.**
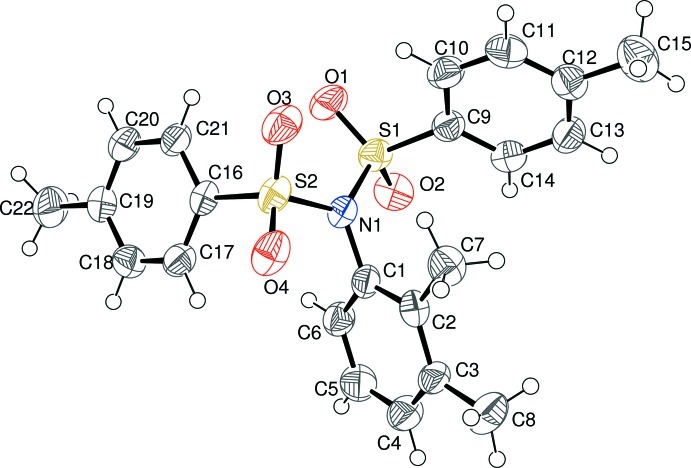
The molecular structure of (I), showing displacement ellipsoids at the 50% probability level.

## supplementary crystallographic information

### Comment

Rather than the intended product of 4-methyl-*N*-(2,3-dimethylphenyl)benzenesulfonamide, the title `double' sulfonamide (in which both amine N—N bonds have been replaced by N—S bonds), (I), (Fig. 1) was unexpectedly prepared. A similar double sulfonamide and previous related structures are described by Mughal *et al.* (2012).

The molecule of (I) cannot possess any local symmetry due to the two methyl groups of the xylene ring. If these are neglected, the rest of the molecule possesses approximate local twofold symmetry about the C1—N1 axis. The dihedral angles between the *o*-xylene ring (C1–C6) and the C9–C14 and C15–C20 toluyl rings are 41.19 (15) and 20.50 (17)°, respectively; the dihedral angle between the toluyl rings is 48.11 (14)°. The sulfonamide torsion angles in (I) are -87.6 (2)(2)° for C1—N1—S1—C9 and 77.43 (18)° for C1—N1—S2—C16. The bond-angle sum for N1 in (I) of 359.4° implies *sp*^2^ hybridization for the N atom. However, its (presumed) unhybridized p orbital is almost orthogonal to the aromatic π system of the C1–C6 ring and the C1—N1 bond length of 1.452 (3) Å is that expected for a C—N single bond.

The only direction interactions in the crystal of (I) are a possible very weak C—H···π bond (Table 1) and aromatic π–π stacking between inversion-related pairs of C9–C14 rings [centroid–centroid separation = 4.010 (2) Å; slippage = 1.987 Å].

### Experimental

0.10 g of 2,3-dimethyl aniline was dissolved in 15 ml dichloromethane and 0.157 g of toulene sulfonyl chloride was added. The mixture was stirred at room temperature overnight and the pH was maintained at 8–9 with triethylamine. On completion of the reaction (after TLC), 1 *M* HCl solution was added and the organic layer was separated and allowed to evaporate at room temperature to generate light-brown crystals in 97% yield. Yellow blocks were recrystallized from ethanol solution.

### Refinement

The H atoms were placed in calculated positions (C—H = 0.93–0.96 Å) and refined as riding. The constraint *U*_iso_(H) = 1.2*U*_eq_(C) or 1.5*U*_eq_(methyl C) was applied. The methyl groups were allowed to rotate, but not to tip, to best fit the electron density.

### Crystal data

**Table:**

C_22_H_23_NO_4_S_2_	*Z* = 2
*M**_r_* = 429.53	*F*(000) = 452
Triclinic, *P*1	*D*_x_ = 1.389 Mg m^−^^3^
Hall symbol: -P 1	Mo *K*α radiation, λ = 0.71073 Å
*a* = 7.5523 (8) Å	Cell parameters from 185 reflections
*b* = 8.4635 (9) Å	θ = 3.3–19.5°
*c* = 17.5004 (17) Å	µ = 0.29 mm^−^^1^
α = 103.282 (7)°	*T* = 296 K
β = 91.659 (7)°	Block, yellow
γ = 108.382 (7)°	0.32 × 0.23 × 0.12 mm
*V* = 1026.89 (18) Å^3^	

### Data collection

**Table:**

Bruker APEXII CCD diffractometer	4569 independent reflections
Radiation source: fine-focus sealed tube	2359 reflections with *I* > 2σ(*I*)
Graphite monochromator	*R*_int_ = 0.068
ω scans	θ_max_ = 27.3°, θ_min_ = 1.2°
Absorption correction: multi-scan (*SADABS*; Bruker, 2007)	*h* = −9→9
*T*_min_ = 0.913, *T*_max_ = 0.966	*k* = −10→10
16232 measured reflections	*l* = −22→22

### Refinement

**Table:**

Refinement on *F*^2^	Primary atom site location: structure-invariant direct methods
Least-squares matrix: full	Secondary atom site location: difference Fourier map
*R*[*F*^2^ > 2σ(*F*^2^)] = 0.055	Hydrogen site location: inferred from neighbouring sites
*wR*(*F*^2^) = 0.129	H-atom parameters constrained
*S* = 0.96	*w* = 1/[σ^2^(*F*_o_^2^) + (0.052*P*)^2^] where *P* = (*F*_o_^2^ + 2*F*_c_^2^)/3
4569 reflections	(Δ/σ)_max_ = 0.002
266 parameters	Δρ_max_ = 0.24 e Å^−^^3^
0 restraints	Δρ_min_ = −0.30 e Å^−^^3^

### Special details

**Table:**

Geometry. All e.s.d.'s (except the e.s.d. in the dihedral angle between two l.s. planes) are estimated using the full covariance matrix. The cell e.s.d.'s are taken into account individually in the estimation of e.s.d.'s in distances, angles and torsion angles; correlations between e.s.d.'s in cell parameters are only used when they are defined by crystal symmetry. An approximate (isotropic) treatment of cell e.s.d.'s is used for estimating e.s.d.'s involving l.s. planes.
Refinement. Refinement of *F*^2^ against ALL reflections. The weighted *R*-factor *wR* and goodness of fit *S* are based on *F*^2^, conventional *R*-factors *R* are based on *F*, with *F* set to zero for negative *F*^2^. The threshold expression of *F*^2^ > σ(*F*^2^) is used only for calculating *R*-factors(gt) *etc*. and is not relevant to the choice of reflections for refinement. *R*-factors based on *F*^2^ are statistically about twice as large as those based on *F*, and *R*-factors based on ALL data will be even larger.

### Fractional atomic coordinates and isotropic or equivalent isotropic displacement parameters (Å^2^)

**Table:**

	*x*	*y*	*z*	*U*_iso_*/*U*_eq_	
C1	0.1812 (4)	0.1458 (4)	0.21323 (18)	0.0419 (8)	
C2	0.1984 (4)	−0.0039 (4)	0.22706 (18)	0.0439 (8)	
C3	0.2906 (4)	−0.0938 (4)	0.17351 (19)	0.0462 (8)	
C4	0.3599 (5)	−0.0318 (4)	0.1105 (2)	0.0569 (9)	
H4	0.4194	−0.0923	0.0751	0.068*	
C5	0.3422 (5)	0.1202 (5)	0.0990 (2)	0.0580 (10)	
H5	0.3914	0.1611	0.0565	0.070*	
C6	0.2535 (5)	0.2086 (4)	0.14952 (18)	0.0477 (8)	
H6	0.2410	0.3099	0.1418	0.057*	
C7	0.1242 (5)	−0.0688 (4)	0.29584 (18)	0.0546 (9)	
H7A	0.0585	0.0028	0.3239	0.082*	
H7B	0.2266	−0.0663	0.3305	0.082*	
H7C	0.0398	−0.1848	0.2776	0.082*	
C8	0.3123 (5)	−0.2576 (4)	0.1842 (2)	0.0694 (11)	
H8A	0.3848	−0.2974	0.1447	0.104*	
H8B	0.1906	−0.3432	0.1789	0.104*	
H8C	0.3753	−0.2372	0.2357	0.104*	
C9	0.2566 (4)	0.3436 (4)	0.41450 (18)	0.0433 (8)	
C10	0.1242 (5)	0.3167 (4)	0.46751 (19)	0.0514 (9)	
H10	0.0154	0.3442	0.4620	0.062*	
C11	0.1568 (5)	0.2491 (4)	0.52790 (19)	0.0570 (9)	
H11	0.0679	0.2307	0.5635	0.068*	
C12	0.3164 (5)	0.2071 (4)	0.53832 (19)	0.0500 (9)	
C13	0.4476 (5)	0.2369 (4)	0.48554 (19)	0.0533 (9)	
H13	0.5568	0.2104	0.4918	0.064*	
C14	0.4203 (5)	0.3057 (4)	0.42331 (18)	0.0468 (8)	
H14	0.5100	0.3259	0.3882	0.056*	
C15	0.3467 (5)	0.1285 (4)	0.6041 (2)	0.0719 (11)	
H15A	0.3366	0.2009	0.6536	0.108*	
H15B	0.4694	0.1175	0.6046	0.108*	
H15C	0.2535	0.0169	0.5961	0.108*	
C16	−0.1829 (4)	0.3224 (4)	0.18865 (18)	0.0419 (8)	
C17	−0.1586 (4)	0.2853 (4)	0.10925 (19)	0.0483 (8)	
H17	−0.1287	0.1878	0.0862	0.058*	
C18	−0.1790 (4)	0.3935 (4)	0.06515 (19)	0.0504 (9)	
H18	−0.1608	0.3694	0.0119	0.061*	
C19	−0.2260 (4)	0.5380 (4)	0.09775 (19)	0.0487 (8)	
C20	−0.2563 (4)	0.5687 (4)	0.17601 (19)	0.0507 (9)	
H20	−0.2927	0.6628	0.1984	0.061*	
C21	−0.2338 (4)	0.4631 (4)	0.22206 (19)	0.0451 (8)	
H21	−0.2528	0.4867	0.2752	0.054*	
C22	−0.2436 (5)	0.6588 (4)	0.0491 (2)	0.0703 (11)	
H22A	−0.3473	0.6978	0.0634	0.105*	
H22B	−0.2646	0.5998	−0.0059	0.105*	
H22C	−0.1301	0.7558	0.0587	0.105*	
S1	0.21539 (12)	0.42043 (10)	0.33376 (5)	0.0459 (2)	
S2	−0.15010 (12)	0.18724 (10)	0.24649 (5)	0.0463 (3)	
O1	0.1014 (3)	0.5258 (3)	0.35325 (13)	0.0608 (7)	
O2	0.3882 (3)	0.4819 (3)	0.30225 (12)	0.0556 (6)	
O3	−0.2238 (3)	0.2258 (3)	0.31952 (13)	0.0610 (7)	
O4	−0.2068 (3)	0.0159 (3)	0.19840 (14)	0.0591 (7)	
N1	0.0840 (3)	0.2420 (3)	0.26533 (14)	0.0391 (6)	

### Atomic displacement parameters (Å^2^)

**Table:**

	*U*^11^	*U*^22^	*U*^33^	*U*^12^	*U*^13^	*U*^23^
C1	0.0376 (19)	0.0415 (19)	0.0474 (19)	0.0163 (16)	−0.0025 (16)	0.0090 (16)
C2	0.0392 (19)	0.0467 (19)	0.0449 (19)	0.0117 (16)	−0.0030 (15)	0.0144 (16)
C3	0.036 (2)	0.051 (2)	0.051 (2)	0.0206 (17)	0.0014 (16)	0.0036 (17)
C4	0.051 (2)	0.060 (2)	0.060 (2)	0.0245 (19)	0.0056 (19)	0.0086 (19)
C5	0.055 (2)	0.073 (3)	0.052 (2)	0.024 (2)	0.0149 (19)	0.023 (2)
C6	0.050 (2)	0.052 (2)	0.049 (2)	0.0225 (18)	0.0090 (17)	0.0189 (17)
C7	0.060 (2)	0.049 (2)	0.056 (2)	0.0148 (18)	0.0050 (18)	0.0190 (18)
C8	0.073 (3)	0.061 (2)	0.086 (3)	0.038 (2)	0.010 (2)	0.018 (2)
C9	0.046 (2)	0.0410 (19)	0.0442 (19)	0.0171 (16)	0.0063 (16)	0.0090 (16)
C10	0.054 (2)	0.058 (2)	0.051 (2)	0.0271 (19)	0.0175 (18)	0.0163 (18)
C11	0.062 (3)	0.064 (2)	0.047 (2)	0.020 (2)	0.0189 (19)	0.0160 (19)
C12	0.057 (2)	0.042 (2)	0.047 (2)	0.0137 (17)	0.0012 (18)	0.0095 (17)
C13	0.052 (2)	0.050 (2)	0.060 (2)	0.0246 (18)	0.0004 (19)	0.0097 (19)
C14	0.045 (2)	0.0447 (19)	0.048 (2)	0.0145 (17)	0.0085 (17)	0.0073 (16)
C15	0.089 (3)	0.064 (3)	0.062 (2)	0.020 (2)	0.000 (2)	0.024 (2)
C16	0.0370 (19)	0.0437 (19)	0.051 (2)	0.0186 (16)	0.0045 (16)	0.0171 (16)
C17	0.047 (2)	0.046 (2)	0.053 (2)	0.0212 (17)	0.0038 (17)	0.0080 (17)
C18	0.050 (2)	0.060 (2)	0.0431 (19)	0.0199 (19)	0.0062 (17)	0.0125 (18)
C19	0.044 (2)	0.053 (2)	0.053 (2)	0.0193 (18)	0.0010 (17)	0.0174 (18)
C20	0.056 (2)	0.047 (2)	0.058 (2)	0.0279 (18)	0.0057 (18)	0.0152 (18)
C21	0.042 (2)	0.052 (2)	0.049 (2)	0.0246 (17)	0.0092 (16)	0.0157 (17)
C22	0.084 (3)	0.078 (3)	0.068 (3)	0.043 (2)	0.008 (2)	0.034 (2)
S1	0.0543 (6)	0.0368 (5)	0.0494 (5)	0.0175 (4)	0.0080 (4)	0.0127 (4)
S2	0.0412 (5)	0.0448 (5)	0.0622 (6)	0.0189 (4)	0.0124 (4)	0.0242 (5)
O1	0.0785 (18)	0.0464 (14)	0.0692 (16)	0.0382 (14)	0.0113 (14)	0.0123 (12)
O2	0.0524 (15)	0.0477 (14)	0.0604 (15)	0.0034 (12)	0.0131 (12)	0.0193 (12)
O3	0.0601 (16)	0.0763 (17)	0.0700 (16)	0.0367 (14)	0.0329 (14)	0.0406 (14)
O4	0.0485 (15)	0.0355 (13)	0.0910 (18)	0.0114 (11)	0.0001 (13)	0.0159 (13)
N1	0.0349 (15)	0.0394 (15)	0.0456 (15)	0.0154 (13)	0.0042 (12)	0.0112 (13)

### Geometric parameters (Å, º)

**Table:**

C1—C2	1.387 (4)	C13—C14	1.386 (4)
C1—C6	1.396 (4)	C13—H13	0.9300
C1—N1	1.452 (3)	C14—H14	0.9300
C2—C3	1.409 (4)	C15—H15A	0.9600
C2—C7	1.487 (4)	C15—H15B	0.9600
C3—C4	1.374 (4)	C15—H15C	0.9600
C3—C8	1.498 (4)	C16—C21	1.375 (4)
C4—C5	1.393 (4)	C16—C17	1.383 (4)
C4—H4	0.9300	C16—S2	1.758 (3)
C5—C6	1.356 (4)	C17—C18	1.365 (4)
C5—H5	0.9300	C17—H17	0.9300
C6—H6	0.9300	C18—C19	1.383 (4)
C7—H7A	0.9600	C18—H18	0.9300
C7—H7B	0.9600	C19—C20	1.374 (4)
C7—H7C	0.9600	C19—C22	1.506 (4)
C8—H8A	0.9600	C20—C21	1.377 (4)
C8—H8B	0.9600	C20—H20	0.9300
C8—H8C	0.9600	C21—H21	0.9300
C9—C14	1.385 (4)	C22—H22A	0.9600
C9—C10	1.385 (4)	C22—H22B	0.9600
C9—S1	1.744 (3)	C22—H22C	0.9600
C10—C11	1.362 (4)	S1—O1	1.419 (2)
C10—H10	0.9300	S1—O2	1.423 (2)
C11—C12	1.379 (4)	S1—N1	1.689 (3)
C11—H11	0.9300	S2—O3	1.419 (2)
C12—C13	1.379 (4)	S2—O4	1.422 (2)
C12—C15	1.500 (4)	S2—N1	1.682 (2)
			
C2—C1—C6	122.1 (3)	C13—C14—H14	120.7
C2—C1—N1	120.2 (3)	C12—C15—H15A	109.5
C6—C1—N1	117.7 (2)	C12—C15—H15B	109.5
C1—C2—C3	117.6 (3)	H15A—C15—H15B	109.5
C1—C2—C7	122.0 (3)	C12—C15—H15C	109.5
C3—C2—C7	120.4 (3)	H15A—C15—H15C	109.5
C4—C3—C2	119.9 (3)	H15B—C15—H15C	109.5
C4—C3—C8	119.6 (3)	C21—C16—C17	120.3 (3)
C2—C3—C8	120.5 (3)	C21—C16—S2	120.1 (2)
C3—C4—C5	121.0 (3)	C17—C16—S2	119.5 (2)
C3—C4—H4	119.5	C18—C17—C16	119.3 (3)
C5—C4—H4	119.5	C18—C17—H17	120.4
C6—C5—C4	120.2 (3)	C16—C17—H17	120.4
C6—C5—H5	119.9	C17—C18—C19	121.5 (3)
C4—C5—H5	119.9	C17—C18—H18	119.2
C5—C6—C1	119.2 (3)	C19—C18—H18	119.2
C5—C6—H6	120.4	C20—C19—C18	118.1 (3)
C1—C6—H6	120.4	C20—C19—C22	120.7 (3)
C2—C7—H7A	109.5	C18—C19—C22	121.1 (3)
C2—C7—H7B	109.5	C19—C20—C21	121.4 (3)
H7A—C7—H7B	109.5	C19—C20—H20	119.3
C2—C7—H7C	109.5	C21—C20—H20	119.3
H7A—C7—H7C	109.5	C16—C21—C20	119.2 (3)
H7B—C7—H7C	109.5	C16—C21—H21	120.4
C3—C8—H8A	109.5	C20—C21—H21	120.4
C3—C8—H8B	109.5	C19—C22—H22A	109.5
H8A—C8—H8B	109.5	C19—C22—H22B	109.5
C3—C8—H8C	109.5	H22A—C22—H22B	109.5
H8A—C8—H8C	109.5	C19—C22—H22C	109.5
H8B—C8—H8C	109.5	H22A—C22—H22C	109.5
C14—C9—C10	120.9 (3)	H22B—C22—H22C	109.5
C14—C9—S1	119.0 (2)	O1—S1—O2	120.61 (13)
C10—C9—S1	120.1 (2)	O1—S1—N1	106.68 (13)
C11—C10—C9	118.6 (3)	O2—S1—N1	105.80 (12)
C11—C10—H10	120.7	O1—S1—C9	109.75 (14)
C9—C10—H10	120.7	O2—S1—C9	108.68 (14)
C10—C11—C12	122.5 (3)	N1—S1—C9	103.99 (13)
C10—C11—H11	118.7	O3—S2—O4	120.69 (14)
C12—C11—H11	118.7	O3—S2—N1	107.89 (14)
C11—C12—C13	118.0 (3)	O4—S2—N1	104.27 (12)
C11—C12—C15	121.5 (3)	O3—S2—C16	109.27 (13)
C13—C12—C15	120.5 (3)	O4—S2—C16	108.54 (14)
C12—C13—C14	121.4 (3)	N1—S2—C16	105.04 (12)
C12—C13—H13	119.3	C1—N1—S2	118.5 (2)
C14—C13—H13	119.3	C1—N1—S1	117.88 (19)
C9—C14—C13	118.6 (3)	S2—N1—S1	123.06 (14)
C9—C14—H14	120.7		
			
C6—C1—C2—C3	−0.8 (5)	C17—C16—C21—C20	−1.4 (5)
N1—C1—C2—C3	178.6 (3)	S2—C16—C21—C20	179.1 (2)
C6—C1—C2—C7	178.9 (3)	C19—C20—C21—C16	−1.1 (5)
N1—C1—C2—C7	−1.7 (5)	C14—C9—S1—O1	−151.8 (3)
C1—C2—C3—C4	0.2 (5)	C10—C9—S1—O1	30.0 (3)
C7—C2—C3—C4	−179.5 (3)	C14—C9—S1—O2	−18.0 (3)
C1—C2—C3—C8	−179.2 (3)	C10—C9—S1—O2	163.8 (3)
C7—C2—C3—C8	1.1 (5)	C14—C9—S1—N1	94.4 (3)
C2—C3—C4—C5	0.6 (5)	C10—C9—S1—N1	−83.8 (3)
C8—C3—C4—C5	−179.9 (3)	C21—C16—S2—O3	12.9 (3)
C3—C4—C5—C6	−0.9 (6)	C17—C16—S2—O3	−166.5 (3)
C4—C5—C6—C1	0.3 (5)	C21—C16—S2—O4	146.4 (3)
C2—C1—C6—C5	0.6 (5)	C17—C16—S2—O4	−33.1 (3)
N1—C1—C6—C5	−178.9 (3)	C21—C16—S2—N1	−102.6 (3)
C14—C9—C10—C11	−1.2 (5)	C17—C16—S2—N1	78.0 (3)
S1—C9—C10—C11	177.0 (3)	C2—C1—N1—S2	−87.0 (3)
C9—C10—C11—C12	0.2 (5)	C6—C1—N1—S2	92.5 (3)
C10—C11—C12—C13	0.7 (5)	C2—C1—N1—S1	101.4 (3)
C10—C11—C12—C15	−178.4 (3)	C6—C1—N1—S1	−79.1 (3)
C11—C12—C13—C14	−0.6 (5)	O3—S2—N1—C1	149.88 (19)
C15—C12—C13—C14	178.5 (3)	O4—S2—N1—C1	20.4 (2)
C10—C9—C14—C13	1.2 (5)	C16—S2—N1—C1	−93.7 (2)
S1—C9—C14—C13	−176.9 (2)	O3—S2—N1—S1	−39.04 (18)
C12—C13—C14—C9	−0.4 (5)	O4—S2—N1—S1	−168.49 (14)
C21—C16—C17—C18	2.4 (5)	C16—S2—N1—S1	77.43 (18)
S2—C16—C17—C18	−178.1 (2)	O1—S1—N1—C1	156.45 (19)
C16—C17—C18—C19	−0.9 (5)	O2—S1—N1—C1	26.9 (2)
C17—C18—C19—C20	−1.5 (5)	C9—S1—N1—C1	−87.6 (2)
C17—C18—C19—C22	178.5 (3)	O1—S1—N1—S2	−14.67 (18)
C18—C19—C20—C21	2.5 (5)	O2—S1—N1—S2	−144.26 (15)
C22—C19—C20—C21	−177.5 (3)	C9—S1—N1—S2	101.31 (17)

### Hydrogen-bond geometry (Å, º)

Cg is the centroid of the C16–C21 ring.

**Table:**

*D*—H···*A*	*D*—H	H···*A*	*D*···*A*	*D*—H···*A*
C8—H8*B*···*Cg*^i^	0.96	2.96	3.912 (4)	169

Symmetry code: (i) *x*, *y*−1, *z*.
